# Listen to the patients! Identifying CML patients' needs analyzing patient-generated content with AI-driven methodologies

**DOI:** 10.3389/fdgth.2023.1243215

**Published:** 2023-12-05

**Authors:** Stefanie Scholz, Isabell Berns, Christian Winkler

**Affiliations:** ^1^ Data Science in Social Economy, SRH Wilhelm Loehe University of Applied Sciences, Fuerth, Germany; ^2^ Health Economics, University of Bayreuth, Bayreuth, Germany; ^3^ AI-driven User Experience Optimization, Nuremberg Institute of Technology, University of Applied Sciences, Nuremberg, Germany

**Keywords:** patient engagement, patient needs, user generated content, chronic myeloid leukemia, artificial intelligence, topic modeling

## Abstract

**Background:**

Various patient support programs exist to provide successful therapy options for patients. Pharmaceutical companies are increasingly recognizing the importance of actively supporting patients in their long-term treatment. In order to effectively assist patients, it is crucial to understand their current needs by taking a look at the patients' opinions.

**Objective:**

This study focuses specifically on chronic myeloid leukemia (CML) and aims to determine if the current patient engagement offerings from pharmaceutical companies adequately address the needs of CML patients. To achieve this, the study uses content generated by CML patients to assess the patient engagement strategies of selected pharmaceutical companies, explore the relevance of medication, their products, and services, and analyze key concerns from the perspective of the patients.

**Methods:**

To address the research questions, various methodologies were employed. Initially, desk research was conducted to identify relevant pharmaceutical companies and internet forums related to CML. Subsequently, content generated by patients was acquired and AI-driven techniques such as topic modeling and topic evolution analyses were used to examine this user-generated content (UGC) within the identified public forums. This involved analyzing topic models and tracking topic changes over time.

**Results:**

The desk research revealed that pharmaceutical companies primarily offer information about the disease and available treatment options. The UGC analysis confirmed the significant role played by the industry in supporting CML patients. Key areas of interest for patients include the disease itself, potential treatment methods and associated side effects, dosage of active substances, and the possibility of switching therapies due to treatment failure or resistance. Stem cell transplantation was also discussed.

**Conclusions:**

Overall, the pharmaceutical industry adequately addresses the needs of CML patients. However, there is room for improvement in educating patients about treatment options, drugs, and their side effects. Psychological support should not be neglected. Since CML patients frequently engage with clinical trial outcomes, there is potential for increased patient involvement in such trials. Further research in this area is recommended.

## 1. Introduction

### 1.1. Background

The landscape of the healthcare market is constantly evolving, leading to significant changes within the pharmaceutical industry. No longer solely viewed as manufacturers of pharmaceuticals, pharmaceutical companies are embracing a new understanding of their role as healthcare providers (1).

While pharmaceutical companies generally offer extensive patient engagement initiatives for commonly occurring diseases, this paper focuses on investigating their patient engagement efforts specifically tailored for CML, a relatively rare disease. Patient empowerment holds particular significance in chronic illnesses like CML, as it necessitates lifelong therapy, with the success of treatment heavily reliant on patients' motivation and active participation in their own care. In order to foster patient self-management, there is a growing call for pharmaceutical companies to assume the role of healthcare providers and develop robust patient engagement strategies tailored to CML patients. These initiatives aim to facilitate access to vital information and support self-care among individuals affected by CML (2).

### 1.2. Objective of the work and research question

This research paper aims to achieve several objectives that are essential for understanding and addressing the needs of patients with CML. Firstly, it examines the resources provided by selected pharmaceutical companies to support CML patients in managing their own care effectively. Understanding the current needs of individuals affected by CML is crucial for making a valuable contribution to their ongoing treatment and well-being. In addition, this study investigates the extent to which Patient Engagement has been embraced within the pharmaceutical industry from both a professional and institutional standpoint. By comparing these findings with insights gained from different perspectives, the research aims to answer the following important questions:

What is the approach of pharmaceutical companies in terms of patient engagement? How do they position themselves in this regard?
To what extent do pharmaceutical manufacturers, their products, or services play a role in selected forums dedicated to the CML community?
From the patient's perspective, what are the key issues and concerns that can be identified?
From the perspective of the pharmaceutical industry, are the relevant topics of patient engagement adequately addressed?

Ultimately, the central research question guiding this study is as follows:

Are the current patient engagement activities of pharmaceutical companies effectively meeting the needs of individuals diagnosed with CML?

### 1.3. Classification of the subject

CML is one of the four forms of leukemia that can affect all age groups, including young people. It can be assigned to the group Myeloproliferative Neoplasms and is a clonal disease of the hematopoietic stem cells of the bone marrow. Typical for CML is the Philadelphia chromosome. The fusion gene BCR-ABL is responsible for the uncontrolled proliferation of white blood cells (3).

The course of CML can be divided into three phases: the chronic phase, the accelerated phase, and the blast crisis (4). These differ essentially in the number of white blood cell progenitors (blasts) (5).

Current therapies aim, among other things, to prevent progression to the accelerated phase or blast crisis (6). According to the latest ELN recommendations, CML therapy is generally performed by regular oral administration of tyrosine kinase inhibitors (TKI). The following substances are approved in Germany: Imatinib, nilotinib, dasatinib, bosutinib and ponatinib (7). *First-line therapy* is always by a TKI. Four substances are already approved for this purpose: Imatinib, the first-generation TKI, and the second-generation TKIs nilotinib, dasatinib, and bosutinib. If resistance or intolerance occurs, a TKI is also chosen in *second-line therapy*. Ponatinib, the third-generation TKI is only used when therapy fails with a first- or second-generation TKI or when a resistant mutation (T315I mutation) is present (7). However, allogeneic stem cell transplantation remains relevant for the therapy of blast crisis (8).

### 1.4. Patient engagement—a definition of terms

As early as 2010, Gruman et al. reported that there is no universally accepted definition of “patient engagement” in the literature. Generally, the concept includes the idea that patients are actively involved in their own health care (9). Also in 2017, Higgins et al. found that “patient engagement” is a widely used term across health care disciplines, but there is no common, basic definition. For this reason, Higgins et al. conducted a concept analysis of the scientific literature with the goal of exploring relevant attributes related to the use of the term “patient engagement” (10). The following attributes were identified: Personalization, Access, Readiness, and the Therapeutic Relationship. In the remainder of the paper, Patient Engagement is defined as follows:

[…] the concept of patient engagement can be defined as the desire and capability to actively choose to participate in care in a way uniquely appropriate to the individual in cooperation with a healthcare provider or institution for the purposes of maximizing outcomes or experiences of care (10).

Again, it is clear that the patient engagement concept is about active participation in one's own care. This understanding of the term is used in this work.

The pharmaceutical industry has recognized the immense value of engaging with patients as customers, moving beyond their traditional focus solely on physicians. Chronic diseases, in particular, offer pharmaceutical companies an opportunity to establish enduring relationships with patients. By promoting education about healthier lifestyles and supporting individuals in self-managing their health, the industry can develop patient-centered strategies that enhance patients' self-assurance in handling their conditions. Consequently, this fosters increased patient involvement and encourages individuals to take a more active role in their healthcare journey (11).

## 2. Methods

### 2.1. Desk research methodology

In the initial phase of this study, a comprehensive secondary research process was employed to identify the pertinent pharmaceutical companies associated with the treatment of CML. The primary objective was to determine the specific drugs and treatment approaches utilized for CML, which subsequently led to insights regarding the pharmaceutical companies involved in related research endeavors. Additionally, an extensive Internet search was conducted to explore the various patient engagement initiatives offered by these pharmaceutical companies for individuals affected by CML. This encompassed a thorough examination of apps, websites, microsites, and online activities orchestrated by the companies. Simultaneously, a meticulous effort was made to identify online forums where CML patients actively participate. Notably, the forums affiliated with leukaemie-online.de, cmlsupport.org.uk, as well as the reddit communities reddit.com/r/leukemia and reddit.com/r/CML were included in the analysis. The inclusion of indication-specific forums like leukaemie-online.de and cmlsupport.org.uk was crucial, while the involvement of the reddit platform was deemed valuable due to its potential for engaging a younger demographic, which tends to be highly active online. This comprehensive approach provided a solid foundation for the subsequent methodology employed in this study.

### 2.2. Methodology of user generated content analysis

By employing the power of artificial intelligence (AI), user-generated content (UGC) from CML patients was subjected to thorough analysis. The data used for this analysis was obtained from publicly available forums identified during the desk research phase. The relevant UGC was carefully downloaded and extracted from these selected sources. The subsequent evaluation of the data was conducted through a series of steps, which will be elaborated upon below.

#### 2.2.1. Topic model methodology

**Figure 1:**
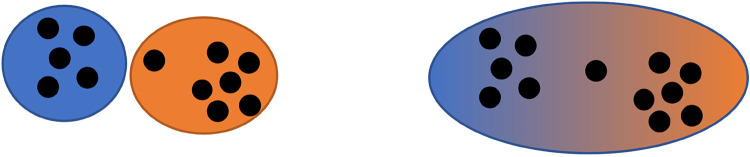
Clustering compared to topic modeling (own illustration).

To gain insights into the underlying structure of a substantial *corpus* consisting of numerous documents, topic modeling (12) was employed. This approach has gained significant popularity over the past decade (13). It can be conceived as a fuzzy version of clustering (see Figure 1). Through this methodology, the latent structure of the corpus was unveiled, and various topics were identified. The primary focus was on uncovering the structural aspects rather than individually assigning documents to specific topics, which could be achieved using clustering, a similar unsupervised machine learning technique.

**Figure 2:**
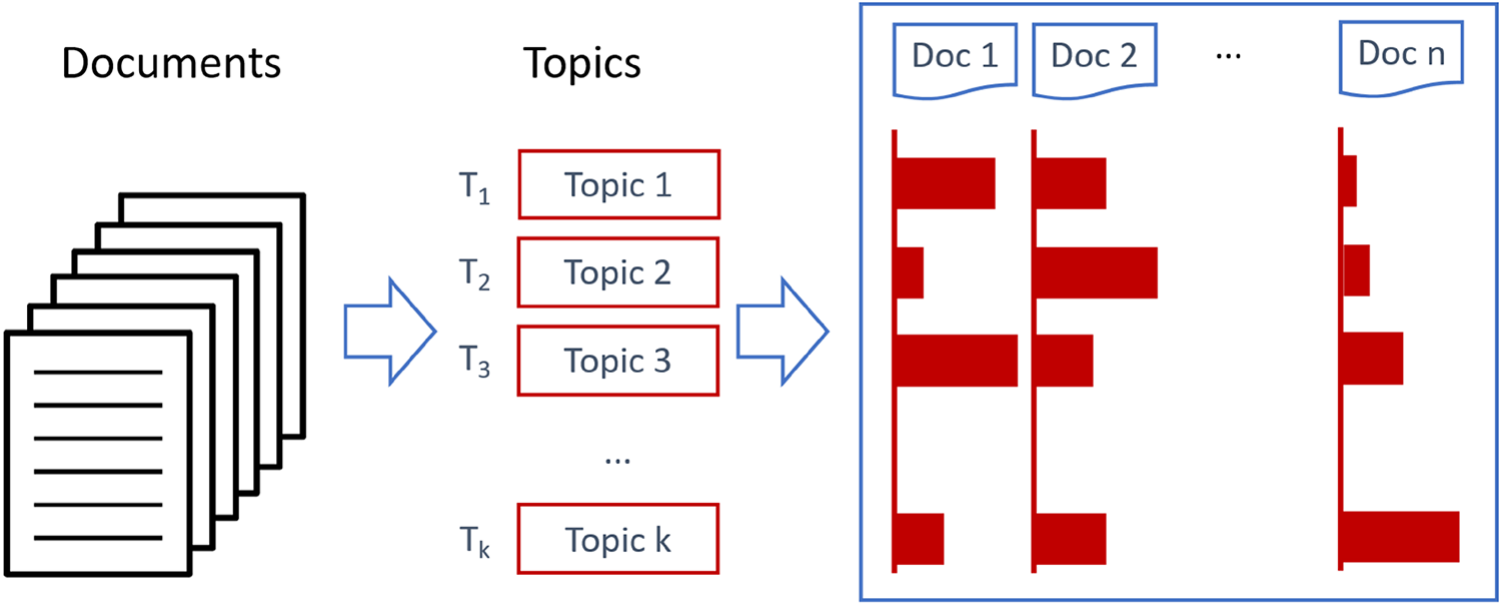
Main tasks of topic modeling (own illustration).

As can be seen in the Figure 2, starting from a large number of documents, the method will find out topics and their contribution to different documents (14). At the same time, the document contribution to the topics will be uncovered. Both relations can be used for a variety of analyses.

This unsupervised method works very well and can be tuned in various dimensions (15). The starting point is the *document-term matrix* which counts how often each word appears in each document. The vocabulary is enumerated, each word gets a number (e.g., in the order of appearance). Stopwords, i.e., words carrying no meaning, can already be eliminated in this step. The order of words is lost; this is a serious restriction and can be taken care of by considering two-word combinations. The document-term matrix contains the words in the columns and the documents in the rows. Its dimensions are (number of documents)×(number of different words). Each cell contains the number of times the word appears in the document. Sometimes, a TF/IDF transformation is applied to penalize words which appear very often in the corpus.

**Figure 3:**
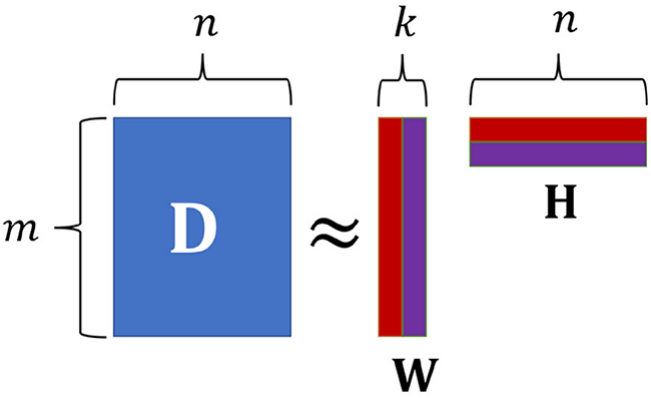
Decomposing a document-term matrix into a document-topic and topic-term matrix (own illustration).

The matrix can become very large and is not suitable for direct interpretation. However, the matrix contains positive entries only (or 0). All such matrices can be decomposed into smaller matrices using k rows/columns where *k* is the rank of the document-term matrix (see Figure 3). An approximation can be applied by choosing a (much) lower *k* which makes both factor matrices interpretable. This is the non-negative matrix factorization method of topic modeling:

In topic modeling, the document-term matrix is usually transposed meaning that it consists of *n* documents and *m* terms.

This method will be applied to the corpus in the next section.

##### 2.2.1.1. Creation of topic models

To gain valuable insights into the prevailing topics within the online community, a qualitative examination was conducted using topic modeling. Since the topics covered by individual websites tend to differ, separate topic modeling was performed for each user-generated content (UGC) source. To facilitate meaningful data interpretation, a maximum of ten topics were generated per website. Visual representation of the topics was provided through word clouds for enhanced qualitative understanding. In order to streamline the analysis and ensure its relevance, stopwords such as “deleted,” “removed,” “thanks,” “thank,” “sharing,” “user,” “https,” “www,” “reddit,” and “com” were pre-emptively removed from all websites. Subsequently, additional stopwords were identified and eliminated from the topic models if they lacked significant semantic value or were deemed irrelevant to the research question at hand. This meticulous approach enhances the clarity and robustness of our findings, allowing for a more compelling and valuable analysis.

##### 2.2.1.2. Analysis of the topic models

In this study, we present an in-depth analysis of topic relevance and word significance within the context of our research. To provide comprehensive insights, we identified 30 subtopics associated with each main topic. The tables presented in this study include topic sizes, which are crucial for assessing the relative importance of individual forums regarding the respective topics. Additionally, we showcase the individual relevance of each word within a topic.

To conduct this analysis, we utilized six Excel files, each containing both unigrams (individual elements) and bigrams (two-word combinations) for every website. These files were meticulously analyzed through an iterative process. To establish a foundation for the subsequent interpretation in our work, we sorted the topics of each website based on their relevance. The weighting of each topic determines its relative importance within the topic models. A lower weighting indicates lower relevance, while a higher weighting signifies greater significance. In our analysis, we assigned numbers to represent the relevance of each topic, with 0 indicating high relevance and 9 representing the lowest relevance within the topic models. By employing this approach, we aim to provide a clear, persuasive, and valuable framework for interpreting the results and drawing meaningful conclusions in the subsequent sections of our research:

→0=veryrelevant,4=relevant,9=lessrelevant.

The weights of the subtopics (terms within a topic) were considered in addition to the topics in order to assign them to categories. Topics that were rated with relevance values from zero to four could be considered for category assignment. Finally, the terms within Topics were examined more closely for feelings or emotions. The terms were assigned to a more positive or a more negative expression.

##### 2.2.1.3. Topic evolution analysis

For the analysis of the time development of the Topic models, diagrams, and the corresponding Excel tables were provided for unigrams with the monthly weights of different years.

For the forum of the German website leukaemie-online.de, data from 01/2010 to 01/2022 were used. For the forum of the website cmlsupport.org.uk, data from 01/2010 to 01/2022 also applied. The data of the two reddit forums reddit.com/r/leukemia and reddit.com/r/CML showed patchy data between the years 2010 up to and including 2014, so that here the January data between 01/2015 to 01/2022 were considered. In this context, the most relevant topic and the least relevant topic were filtered out for each forum and the years of highest and lowest January values were analyzed.

Since the January values are not meaningful for all months within a year and can only be considered as a possible trend, the average values of all months between January 2010 and February 2022 (for leukaemie-online.de) and March 2022 (for cmlsupport.org.uk) were also calculated. For the reddit forums, the average values between January 2015 and March 2022 were calculated.

## 3. Results

### 3.1. Desk research

#### 3.1.1. Patient engagement activities of pharmaceutical companies

During the process of internet research, websites pertaining to CML and other support services were identified.

The findings of the analysis conducted on Novartis' patient engagement efforts are summarized in Table 1. The German website www.leben-mit-cml.de provides a wide range of information about CML. It covers basic inquiries, outlines symptoms, and discusses the latest treatment options for CML patients. Furthermore, the website offers information about upcoming “MPN Patient Days” and allows free registration. It also provides downloadable brochures on different topics like “CML—An Overview,” “Suggestions for Relatives,” and “Questions for the Doctor's Interview.” In addition, the “Medication Plan Creates an Overview” initiative is actively supported by Novartis.

**Table 1:** Patient Engagement offerings from novartis (own illustration).

Information websites	- Initiative “Living with CML • https://www.leben-mit-cml.de/ - Overview of CML • https://www.novartis.com/diseases/chronic-myeloid-leukemia - CML Information graphic • https://www.novartis.com/media-library/understanding-chronic-myeloid-leukemia-infographic - Contribution to Patient Perspectives • https://www.novartis.com/stories/future-chronic-myeloid-leukemia-evolving
Further support	- Patient Days • https://www.mpn-patiententage.de/ - Initiative “Medication plan creates overview • https://www.medikationsplan-schafft-ueberblick.de/ - Website support • https://www.leukaemie-online.de/

By highlighting these important aspects, it is clear that Novartis is actively involved in connecting with patients with CML and offering them information resources and support. The company's indication-specific website, CML-specific brochures, and participation in pertinent initiatives showcase Novartis' commitment to fostering a comprehensive understanding of CML.

The pharmaceutical company Bristol-Myers Squibb also offers informative websites. The website www.krebs.de enables patients to obtain information on various types of cancer, including CML. In particular, the clinical picture, symptoms, diagnosis, staging, and possible therapies are discussed here. Furthermore, the site provides a first insight into the topics “Living with cancer” or in various points regarding questions after the diagnosis (see Table 2).

**Table 2:** Patient Engagement offerings from Bristol-Myers Squibb (own illustration).

Information websites	- CML Overview • https://www.krebs.de/krebsarten/cml - CML Information graphic • https://www.bms.com/media/media-library/disease-state-infographics/chronic-myeloid-leukemia-at-a-glance.html

Pfizer supports patients through informative websites and through the involvement of various initiatives. Table 3 provides an overview. Both the corporate website and the microsite www.gethealthystayhealthy.com address various topics related to CML. To share expertise and help patients manage their own health, various resources developed by Pfizer healthcare professionals are published on the website www.gethealthystayhealthy.com (16).

**Table 3:** Patient engagement offerings from Pfizer (own illustration).

Information websites	- CML Overview • https://www.pfizer.com/news/articles/living_with_chronic_myeloid_leukemia_cml - Land of Health Initiative • https://www.landdergesundheit.de/und-sonst/blutkrebs-viel-erreicht-noch-viel-tun-prof-dr-med-florian-heidel
Further support	- Project “Help for me” • https://www.hilfefuermich.de/ - Initiative “Me at the Doctor's Office"/ “Me in the Hospital • https://www.ichbeimarzt.de/initiative/ https://www.ichbeimarzt.de/ichimkrankenhaus/ - Initiative “Medication plan creates overview • https://www.medikationsplan-schafft-ueberblick.de/

Like Novartis, Pfizer is also involved in the “Medication Plan Creates Overview” initiative. It also offers various projects such as “Me at the doctor's”. Here, tips are provided for the doctor-patient conversation. In addition to patient and senior citizen organizations, external communications experts and Pfizer employees have also contributed.

Compared to the other pharmaceutical companies mentioned, Incyte offers two apps that are tailored to the disease CML (see Table 4). The app “CML Life” has been available in the app store since 2018 and shows over 100 downloads (as of May 2022). The app offers the following functions: Personalized and up-to-date information about CML; CML patient experiences; Podcasts from health psychologists with a focus on well-being in CML; Health tracker, which provides a shareable, personalized digital record of tests and treatment-related statistics; Well-being and health literacy questionnaires that provide information about how CML patients are doing at home and help CML patients gain understanding of the disease and confidence in treatment; and a discussion guide for doctor-patient conversations (17). The “CML Scores” app was released in 2021. It is used to calculate individual risk and assess treatment response for CML sufferers by ELTS, EUTOS or Sokal score (18).

**Table 4:** Patient engagement offerings from Incyte (source: own representation).

Information websites	- Event “CML One • https://cml-one.com/
Further support	- APP “CML Life - APP “CML Scores

In addition, Incyte organizes and sponsors the CML-ONE event. During this event small improvements in the treatment of CML, that can lead to significant progress in patient outcomes. Highlights of the event have been made available on the website www.cml-one.com, which is not available anymore.

#### 3.1.2. Forums

The central issues of CML patients, as well as the their perception of pharmaceutical companies and their products and/or services within the community, form relevant sub-questions of this work. In addition to public forums, the social media platform “Facebook” of the U.S. company “Meta” offers groups for CML sufferers and relatives, but the majority of those forums established are not open to the public. It was also possible to identify other forums on the Internet. Only public forums that do not require further registration and are independent from pharmaceutical companies were considered in this work (see Table 5).

**Table 5:** Overview of internet forums for CML patients (own illustration).

Link^a^	Number of contributions^a^	Language
https://www.leukaemie-online.de/diskussionsforen	≈20.600	German
https://cmlsupport.org.uk/forum	≈46.070	English
https://www.cancerresearchuk.org/about-cancer/cancer-chat/posts?query=CML	≈47.500	English
https://community.macmillan.org.uk/cancer_types/chronic-myelogenous-leukemia-forum	≈80	English
https://forum.bloodcancer.org.uk/	≈930	English
https://www.reddit.com/r/CML/	Without specification	English
https://www.reddit.com/r/cancer/		
https://www.reddit.com/r/leukemia/		

^a^ Status March 2022.

A search engine research showed that there are hardly any public German-language forums. The same research led to many English-speaking CML patient communities, but the forums “CML Support” and “Cancer-Research-UK” stand out in terms of the number of posts. The website www.reddit.com was also found but does not provide any information on the number of posts, but here, too, a lively exchange about CML within the community could be observed.

### 3.2. Results of the user generated content analysis

To answer the sub-questions of whether pharmaceutical companies, their products and services have a significance for the CML community and which central topics CML sufferers address in selected forums, UGC was analyzed. The results of this investigation provide information on relevant topics of those affected, whether these show a positive or negative expression, as well as the development of the main topics over time.

Three Internet forums from Table 5 were selected for the present work. With the help of an AI-supported analysis—different approaches of Natural Language Processing were applied –, user-generated texts from the forums listed in Table 6 were analyzed, which are suitable due to the sufficient number of posts. The two subforums of the website reddit are considered together here.

**Table 6:** Selection of UGC sources (own illustration).

Link^a^	Number of contributions	Language
https://www.leukaemie-online.de/diskussionsforen	≈20.600	German
https://cmlsupport.org.uk/forum	≈108.638	English
https://www.reddit.com/r/CML/	≈40.583	English
https://www.reddit.com/r/leukemia/		

^a^ Status March 2022.

#### 3.2.1. Topic evolution

In the following, the development of the Topic models over the course of the last few years is presented. Since the data of the bigrams are not sufficient, only the data of the unigrams are examined. For the forum of the website leukaemie-online.de, the data from January 2010 to February 2022 are used. The forum of the website cmlsupport.org.uk provides data from January 2010 to March 2022. The data of the two reddit forums reddit.com/r/leukemia and reddit.com/r/CML are considered between January 2015 to March 2022 due to incomplete data.

##### 3.2.1.1. leukaemie-online.de

**Figure 4:**
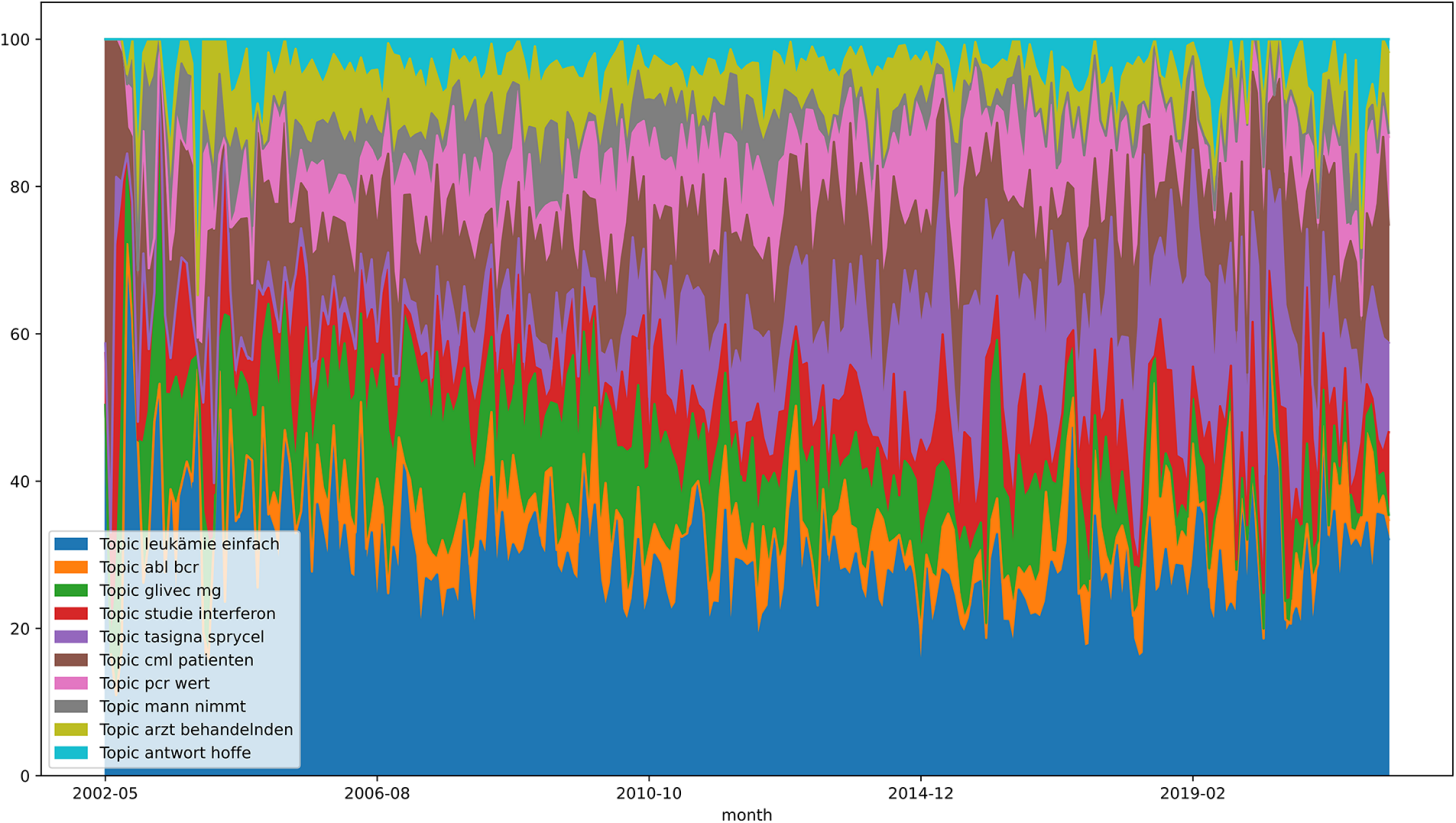
Topic evolution graph—leukaemie-online.de.

If the average values of all months between 01/2010 and 02/2022 are considered, the topic “leukämie einfach” (in English: “leukemia simple”) is the most significant topic with a value of ≈27.476, which is also confirmed in the above result. This is followed by “tasigna sprycel” (≈14.427) and “cml patienten” (in English: “cml patients”) (≈12.952). The smallest average value is carried by “man nimmt” (in English: “man takes”) with a value of ≈3.339, as well as “arzt behandelnden” (in English: “doctor treating”) (≈4.330) and “antwort hoffe” (in English: “answer hope”) (≈5.385) (see Figure 4).

##### 3.2.1.2. Cmlsupport.org.uk

The average weighting values were also calculated here based on all months between 01/2010 and 03/2022. With a value of ≈24.383, it is also confirmed here that the topic “time know” is the most relevant topic of the forum. This is followed by “cml patients” (≈17.047) and “pcr test” (≈11.090). The lowest mean scores are shown by “reply appreciate” (≈2.769), which has already been identified as less relevant, and “vitamin d3” (≈4.366) and “news fantastic” (≈5.150).

**Figure 5:**
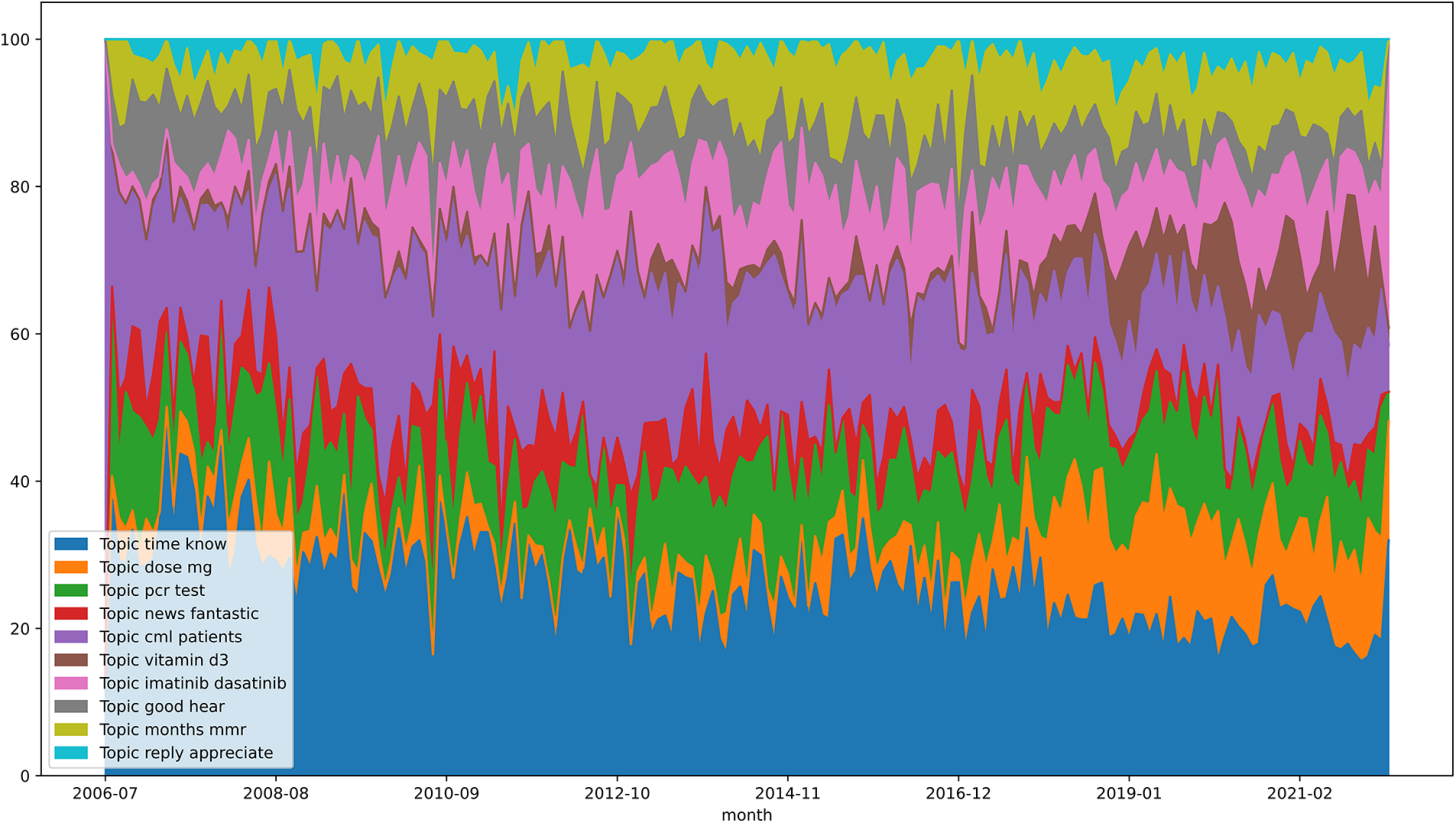
Topic evolution graph—cmlsupport.org.uk.

The following Figure 5, in which “time know” is also the most relevant topic and, as with the average values, is followed by “cml patients”. It can also be seen that the topic “dose mg” has increased since 2018, but especially since 2020.

##### 3.2.1.3. Reddit.com/r/leukemia and reddit.com/r/CML

Since the data between 2010 and 2014 inclusive are incomplete, the data of the forums on the Reddit website are treated from 2015. The average weighting between 01/2015 and 03/2022 also confirms that the topic “like time” (≈28.072) is the most relevant. This is followed by “leukemia symptoms” (≈16.546) and “blood test” (≈9.143). The topics “hope soon” (≈4.986), “doctor ask” (≈5.076), and “know let” (≈6.235) are least strongly weighted.

**Figure 6:**
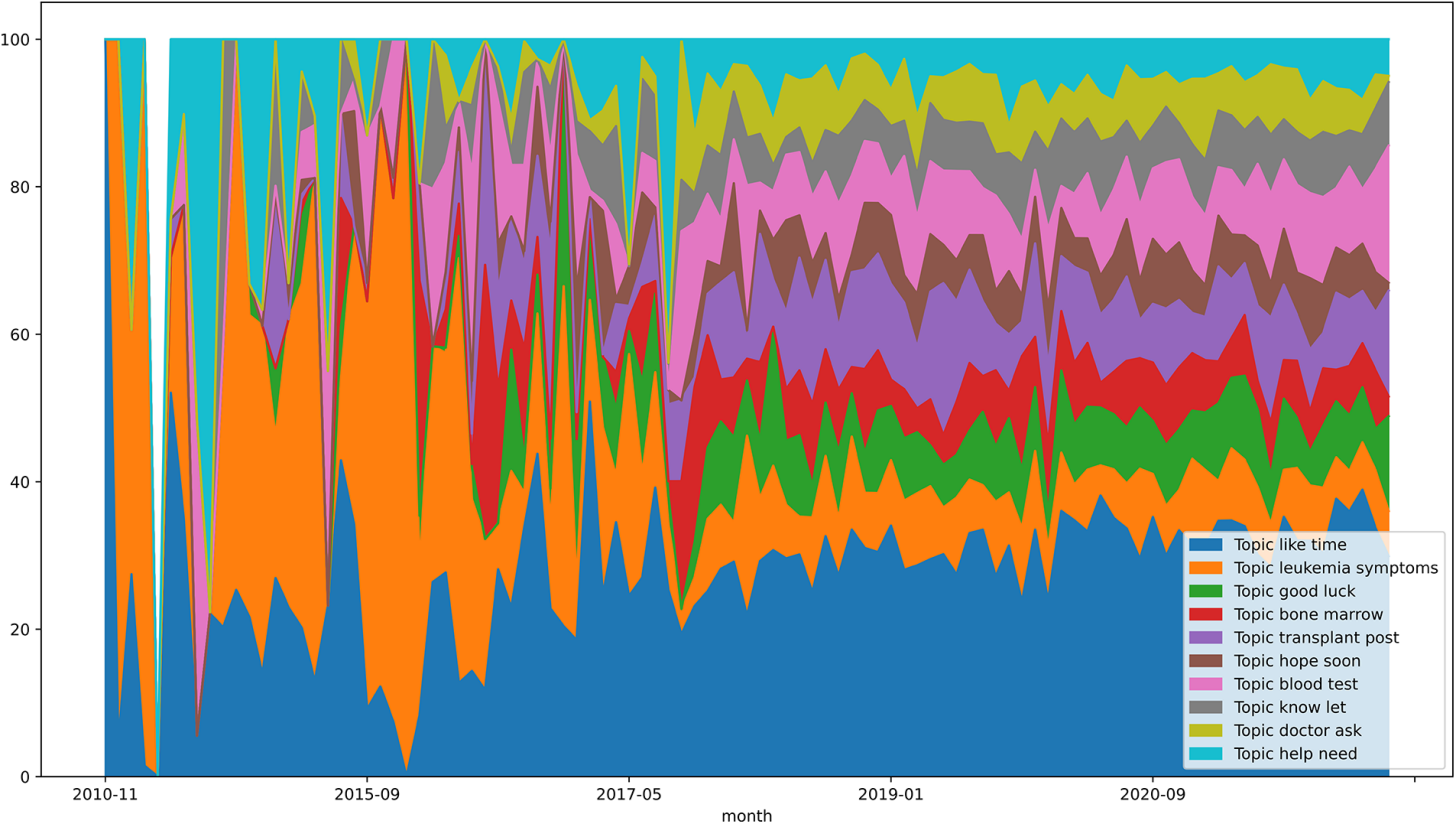
Topic evolution graph—reddit.com/r/leukemia and reddit.com/r/CML.

On the following page, the topic evolution of the two Reddit forums is shown in Figure 6. Here, too, it is clear that “like time” has always been one of the most relevant topics over the years. Likewise, a clear decline in the topic “leukemia symptoms” can be seen from around 2016.

#### 3.2.2. Topic models

**Figure 7:**
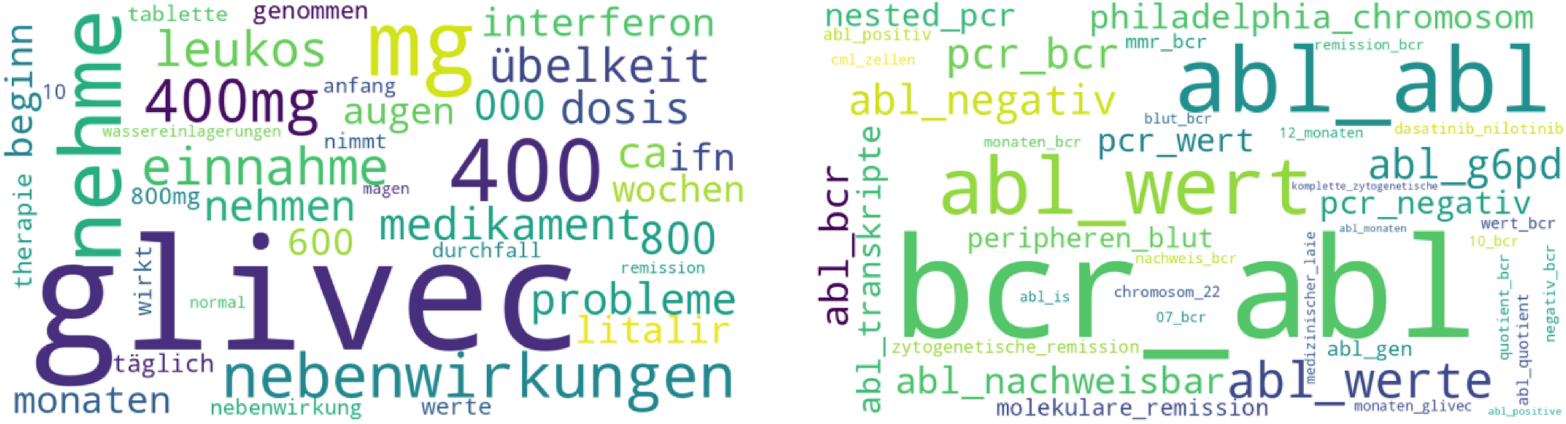
Topic clouds for leukaemie-online.de (left: unigram, right: bigram).

**Figure 8:**
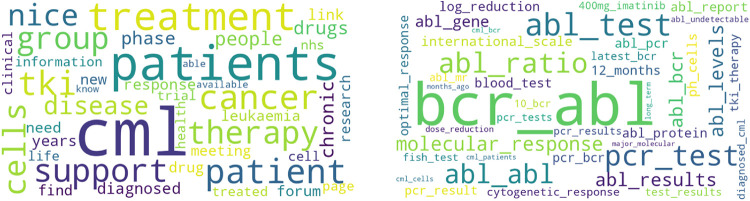
Topic clouds for cmlsupport.org.uk (left: unigram, right: bigram).

**Figure 9:**
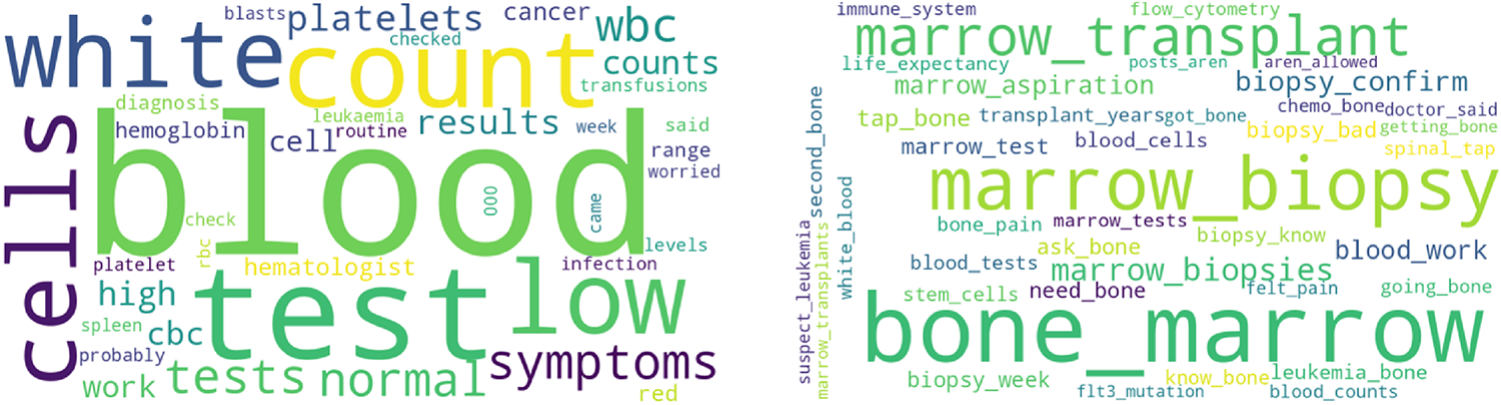
Topic clouds for reddit (left: unigram, right: bigram).

Tables 7–9 provide an overview of the weighting of the uni- and bigrams of the three selected websites. The smaller the weighting (“Size” column), the less relevant the topic is within the present models. For visualization purposes, word clouds are also generated for selected topics (see Figures 7–9, within the Supplementary Material the word clouds and tables of 10 topics for each forum can be found). The larger a word, the more relevant it is for the specific topic.

**Table 7:** Topic relevance overview (Uni & bigram) of leaukaemie-online.de.

Unigram	Size	Relevance	Bigram	Size	Relevance
TOPIC leukämie (00)	0.2826…	0	TOPIC bcr abl (00)	0.2119…	0
TOPIC cml (05)	0.1195…	1	TOPIC 400 mg (01)	0.1353…	1
TOPIC glivec (02)	0.1148…	2	TOPIC nehme glivec (05)	0.1103…	2
TOPIC tasigna (04)	0.1021…	3	TOPIC cml patienten (06)	0.1026…	3
TOPIC pcr (06)	0.0911…	4	TOPIC prof hochhaus (02)	0.1012…	4
TOPIC studie (03)	0.0756…	5	TOPIC nächste woche (09)	0.0781…	5
TOPIC arzt (08)	0.0651…	6	TOPIC wünsche kraft (07)	0.0714…	6
TOPIC abl (01)	0.0604…	7	TOPIC schönen abend (04)	0.0647…	7
TOPIC antwort (09)	0.0407…	8	TOPIC schönes	0.0629…	8
			wochenende (08)		
TOPIC mann (07)	0.0474…	9	TOPIC org wiki (03)	0.0611…	9

**Table 8:** Topic relevance overview (uni & bigram) from cmlsupport.org.uk.

Unigram	Size	Relevance	Bigram	Size	Relevance
TOPIC time (00)	0.2419…	0	TOPIC bcr abl (00)	0.1473…	0
TOPIC cml (04)	0.1643…	1	TOPIC 20 mg (04)	0.1226…	1
TOPIC pcr (02)	0.1142…	2	TOPIC bone marrow (05)	0.1151…	2
TOPIC dose (01)	0.0937…	3	TOPIC let know (03)	0.1096…	3
TOPIC imatinib (06)	0.0925…	4	TOPIC ng ml (06)	0.1057…	4
TOPIC months (08)	0.0847…	5	TOPIC log reduction (02)	0.1004…	5
TOPIC good (07)	0.0724…	6	TOPIC good news (01)	0.0907…	6
TOPIC vitamin (05)	0.0568…	7	TOPIC support group (07)	0.0883…	7
TOPIC news (03)	0.0475…	8	TOPIC nlm nih (08)	0.0737…	8
TOPIC reply (09)	0.0317…	9	TOPIC spaces live (09)	0.0462…	9

**Table 9:** Topic relevance overview (uni & bigram) from reddit.

Unigram	Size	Relevance	Bigram	Size	Relevance
TOPIC like (00)	0.3221…	0	TOPIC bone marrow (00)	0.2023…	0
TOPIC blood (06)	0.0962…	1	TOPIC good luck (01)	0.1327…	1
TOPIC transplant (04)	0.0954…	2	TOPIC stem cell (02)	0.1256…	2
TOPIC leukemia (01)	0.0821…	3	TOPIC post transplant (03)	0.1075…	3
TOPIC good (02)	0.0786…	4	TOPIC years ago (06)	0.0892…	4
TOPIC know (07)	0.0708…	5	TOPIC sounds like (05)	0.0842…	5
TOPIC bone (03)	0.0702…	6	TOPIC blood test (04)	0.0841…	6
TOPIC doctor (08)	0.0656…	7	TOPIC second opinion (08)	0.0629…	7
TOPIC hope (05)	0.0612…	8	TOPIC ask doctor (09)	0.0621…	8
TOPIC help (09)	0.0572…	9	TOPIC mind asking (07)	0.0490…	9

It should be mentioned that in general all topics of this work are relevant. The relevance rating refers exclusively to the raw data. First, the topics of the individual websites are sorted according to their relevance.

In the following tables, the numbers zero to nine express the relevance of the individual topics.

→0=veryrelevant,4=relevant,9=lessrelevant.

In addition to the topic relevance, the weighting of the terms within the topics is also considered. In addition, the topics with relevance values from 0 to 4 are assessed and assigned to categories in the following.

##### 3.2.2.1. leukaemie-online.de

The topics of the uni and bigram with relevance values from 0 to 4 from Table 7 were then grouped into the following categories:

- Category disease: Topic leukämie (English “leukaemia”) (00), Topic cml (05), Topic bcr-abl (00).
- Category therapy: Topic glivec (02), Topic tasigna (04), Topic pcr (06), Topic 400 mg (01), Topic nehme glivec (English “take glivec”) (05).
- Category Exchange/Support: Topic cml patienten (English “cml patients”) (06).
- Category Health Care Professionals: Topic Prof Hochhaus (02).

Figure 7 shows a Word Cloud for the Unigram “glivec” and a Word Cloud for the Bigram “bcr abl” Both topics also appear in Table 7. The size of the individual words shows which words occur with which probability in the specific Topics. The larger a word is, the higher the probability. The topic “glivec” contains aspects such as Einnahme/Nehmen/Nehme (English translation in brackets: “take”), Nebenwirkungen/Übelkeit/Durchfall (“side effects”, “nausea”, “diarrhoea”), Probleme (“problems”), Remission (“remission”), Leukos (“leukocytes”), Augen (“eyes”). The Bigram “bcr abl” contains discussions around topics “abl Wert” (“abl value”), “abl nachweisbar” (“abl provable”), aspects around Remission (“molecular remission”, “cytogenetic remission”).

##### 3.2.2.2. cmlsupport.org.uk

For the forum in cmlsupport.org.uk (see Table 8), the following categories could be identified for the relevance values 0 to 4:

- Category disease: Topic cml (04), Topic bcr-abl (00).
- Category Therapy: Topic pcr (02), Topic dose (01), Topic imatinib (06), Topic 20 mg (04), Topic bone marrow (05), Topic ng ml (06).
- Category Exchange/Support: Topic time (00), Topic let know (03).

Figure 8 shows a Word Cloud for the Unigram “cml” and a Word Cloud for the Bigram “bcr abl”. Both topics also appear in Table 8. As before, the topic “bcr abl” is also the most relevant topic on the website cmlsupport.org.uk. However, if you compare the Word Clouds for the bigram in Figures 7, 8, the individual words vary.

##### 3.2.2.3. Reddit.com/r/leukemia and reddit.com/r/CML

Table 9, with reference to the forums of the website reddit, has the following categories of relevance values 0 to 4:

- Category disease: Topic leukemia (01).
- Category Therapy: Topic blood (06), Topic transplant (04), Topic bone marrow (00), Topic stem cell (02), Topic post transplant (03).
- Category exchange/support: Topic like (00), Topic good (02), Topic good luck (01), Topic years ago (06).

Figure 9 shows a Word Cloud for the Unigram “blood” and a Word Cloud for the Bigram “bone marrow”. Both topics also appear in Table 9.

Overall, the analysis of Tables 7–9 shows that the categories “disease”, “therapy” and “exchange/support” in particular occur in the three forums and are always in the “very relevant” and “relevant” range. Thus, it can be concluded that these categories represent significant topics for CML sufferers and forum members. Particular mention should be made of the “Therapy” category, as this is where the most assignments are made.

In addition to the topic weighting, we also examined whether topics exist in more than one website. A total of eight out of 30 topics exists in two of the three selected Internet forums. German language terms were translated into English where appropriate. For a direct illustration of the weighting, the column “Relevance” represents the values of Tables 7–9.

It can be seen that the topic CML is in the “very relevant” area both in the forum of the website leukaemie-online.de and in the forum of cmlsupport.org.uk. This can also be seen with the topic bcr abl. With a relevance value of zero in two forums, the topic is in the “very relevant” range. The largest difference between the respective values is found in the topic leukemia/leukemia. Nevertheless, with values of zero and three, it is still in the “very relevant” range. Overall, the values of a topic do not differ greatly from one another. This suggests that the topics in the different forums have a similar relevance for forum members. Regarding Table 10, the topic answer/reply with the values eight and nine is the least relevant topic.

**Table 10:** Multiple topics (source: own representation).

LeukemiaOnline	CMLSupport	Reddit		Relevance			
Unigram					L.O^a^	C.S^a^	R.^a^
(00) leukemia	-	(01) leukemia			0		3
-	(07) good	(02) good				6	4
(05) CML	(04) CML	-			1	1	
(06) pcr	(02) pcr	-			4	2	
(08) doctor	-	(08) doctor			6		7
(09) answer	(09) reply	-			8	9	
Bigram							
(00) bcr abl	-	(00) bcr abl			0	0	
-	(00) bone marrow	(05) bone marrow			0	2	

^a^ L.O, Leukaemia Online; C.S, CML Support; R., Reddit.

## 4. Discussion

### 4.1. Desk research

To investigate the positioning of pharmaceutical companies in relation to CML, it was imperative to identify the relevant companies. Through an extensive internet search, Novartis, Bristol-Myers Squibb, Pfizer, and Incyte were identified as the primary companies of interest. While the number of companies may initially appear limited, it is worth considering the rarity of CML as a disease, making this quantity appropriate. Nonetheless, it is possible that other pharmaceutical companies may also play a role, although our intensive research did not uncover them. Notably, Novartis stands out for its readily available offerings, which are easily accessible to the public. On the other hand, Bristol-Myers Squibb and Incyte presented greater challenges in terms of finding their specific offers or services. However, it is important to acknowledge that all mentioned companies do provide information on CML.

Furthermore, we examined the availability of targeted apps developed by pharmaceutical companies. Incyte emerges as the sole company offering two CML apps. Novartis previously had the app “Leben mit CML” (“living with CML”) published in 2014, but it is no longer available in the German App Store. The limited number of downloads for “CML Life” and “CML Scores,” along with the removal of the Novartis app, suggests a low demand for indication-specific apps among CML patients. Potential reasons for this could include insufficient awareness of the apps, a lack of alignment with the needs of CML patients, or simply a lack of interest in utilizing such apps. Notably, the “CML Life” app appears to be highly professional, versatile, and customizable, implying that CML patients either do not perceive a need for its features or remain unaware of its existence. In summary, the first sub-question of this paper can be addressed as follows: Pharmaceutical companies primarily focus on providing information about the disease and therapy options. They accomplish this through their official websites or specialized disease-focused websites. Therefore, it appears that informing patients and/or their families about a particular disease is a significant priority for pharmaceutical companies.

### 4.2. UGC analysis

#### 4.2.1. Topic models

To answer the second sub-question, whether pharmaceutical manufacturers, their products or services play an important role for the CML community in selected forums, and the third sub-question, which central topics can be identified from the patient perspective, it is necessary to look at Tables 7–9 in Chapter 3.2.2 and at the topic models.

In the German-language forum of the website “leukaemie-online.de”, pharmaceutical companies are not directly named. However, the following topics can be associated with pharmaceutical companies, as they relate to active ingredients, dosage or studies:

- For Unigram: *glivec* (Topic 02), *tasigna* (Topic 04), *studie* (Topic 03).
- For bigram: *400 mg* (Topic 01) and *take glivec* (Topic 05).

The topics *glivec*, *tasigna*, *400 mg* and *take glivec* were assigned to the category “therapy”. The relevance of the topics mentioned is in the very relevant to relevant range and thus also expresses a higher importance for the community. The topic *studie* was not assigned to any category, as the relevance value here is five. Nevertheless, it reflects an important topic for those affected.

Looking at the topic model for leukaemie-online.de, it can be seen that topics such as dosage, side effects and problems (e.g., nausea, headaches), tolerability or possible combinations of the drugs are frequently associated with the topics of the active ingredients. Patients report mainly bleeding problems in the eye due to the drug Gleevec. The package insert for Gleevec 100 mg and 400 mg film-coated tablets discusses the side effect “eye pain or worsening of vision, bleeding in the eyes” (19). However, the issue is repeatedly mentioned by the community, so more education may be needed for affected individuals in this case.

In the topic *study,* a possible exchange about studies related to the agents imatinib, nilotinib, dasatinib and TKIs in general can be identified. Furthermore, the TIGER study is mentioned, which investigates the optimization of first-line therapy and is expected to be completed in December 2022 (20). At Topic 02 *prof hochhaus of* the bigram of leukaemie-online.de is the possibility that this is Prof. Dr. med. Andreas Hochhaus, the person responsible for the TIGER study. Both, the expert, and the study seem to be in the interest of the CML community. In addition, the Pegasys trial is also mentioned, which was completed in 2014 (21). As with the active ingredients, side effects and possible combinations are additionally addressed.

In the English-language forum of the website cmlsupport.org.uk, the name of a pharmaceutical company is also not mentioned. However, an indirect reference to the pharmaceutical industry is also recorded here. This results from the following topics:

- For Unigram: *imatinib* (Topic 06)
- For bigram: *20 mg* (Topic 04)*, nlm nih* (Topic 08).

The three topics mentioned were assigned to the “Therapy” category. The relevance values range from very relevant (Topic 4: 20 mg) to less relevant (Topic 8: nlm nih). Topic 6 (imatinib) is exactly in the middle range with a value of four. The Topic imatinib (first-generation TKI) is addressed with second-generation TKIs (dasatinib, nilotinib, and bosutinib) and the third-generation TKI (ponatinib). This indicates an exchange of the community on possible therapy changes or an exchange of experiences with additional agents. Side effects are not directly mentioned in this case but may well be addressed.

The topic nlm nih points to the website www.nlm.nih.gov. This is the National Library of Medicine. Among other things, it offers a database of privately and publicly funded clinical studies and enables active searches for clinical studies. For this reason, this Topic is included in the observation. Looking at the topic model for reddit.com/r/leukemia and r/CML it is noticeable that the topics “nlm nih”, “nih gov” as well as “ncbi nlm” can also be assigned to the mentioned website, so that the assumption is confirmed. The site provides direct access to PubMed, a database of medical articles, which is also mentioned more often by the community. In this context, two articles are directly mentioned, which seem to be relevant within the topic nlm nih. The subtopic “pubmed 29723397” represents an article on PubMed with PubMed ID 29723397, which leads to the study article “Early results of lower dose dasatinib (50 mg daily) as frontline therapy for newly diagnosed chronic-phase chronic myeloid leukemia” (22). The subtopic “pubmed 28396095” leads to the study “Elderly Patients With Chronic Myeloid Leukemia Benefit From a Dasatinib Dose as Low as 20 mg” (19). Both articles involve a lower-dose regimen of the drug dasatinib. It can be assumed that patients have a greater interest in reducing the drug dosage. This can also be justified by the fact that the topic “20 mg” has a very high relevance in the forum of the website www.cmlsupport.org.uk. Overall, it can be seen within the topic nlm nih that forum members exchange more about articles and studies.

In the English-language forums on the website reddit.com, no pharmaceutical company is mentioned directly and no indirect reference to the pharmaceutical industry can be identified via the topics. One explanation for this could be that, in comparison to the websites leukaemie-online.de and cmlsupport.org.uk, reddit is not an indication-specific forum but a website that covers a wide range of topics (20).

*The second sub-question can thus be answered as follows*: The pharmaceutical industry plays an important role for patients with CML. Especially therapy options, here especially drugs, are of great importance. In addition, forum members increasingly discuss clinical studies and medical articles, which are also indirectly attributable to the pharmaceutical industry. When looking at the results, it is also noticeable that topics relating to the disease itself have a high relevance. This indicates that education and knowledge about the disease itself, as well as therapy options, are important topics for CML sufferers.

The central concerns expressed by CML patients primarily revolve around the disease itself, available treatment methods and their associated side effects, appropriate dosage of medications, and the possibility of switching therapies in cases of treatment failure or resistance. Additionally, the topic of stem cell transplantation is discussed. The importance of exchange and support within the CML community is also evident. Surprisingly, the topic models did not reveal any direct or indirect mentions of apps. Furthermore, the existing apps developed by pharmaceutical companies have shown minimal downloads, suggesting a lack of interest in apps within the CML community.

In spite of the contemporary emphasis on attaining treatment-free remission (TFR) as a pivotal goal in the management of CML and its recognition as a viable therapeutic approach according to global guidelines (21, 23), it is noteworthy that analyses of patient-generated discussions do not expose that as a separate topic. Therefore, subsequent analyses were used by determining word frequencies associated with “treatment-free remission,” “therapy discontinuation,” and “TKI withdrawal” within user-generated content across various forums. This revealed a conspicuous absence of these aspects in the discourse. Notably, in the largest forum, www.cmlsupport.org.uk, comprising a substantial total of 108,638 posts, discussions covering TFR and analogous topics accounted for merely 1% of the overall discourse (approximately 1,200 instances). This minimal representation in discussions indicates that these critical aspects are not addressed as standalone topics.

The focus of this study was to determine whether the patient engagement activities of pharmaceutical companies adequately meet the needs of CML patients. To address this question, an inductive comparison was conducted, contrasting the findings from desk research, user-generated content analysis, and patient engagement resources provided by the pharmaceutical industry.

The needs of CML patients are evident based on the results of this study. The analysis of user-generated content revealed that the central concerns of patients revolve around the disease itself, available treatment options including side effects, as well as the importance of community exchange and support. These topics appear particularly relevant, especially for patients at the beginning of their disease journey. However, these topics remained significant throughout the study, suggesting that patients who have been living with the disease for an extended period also have ongoing questions and a need for information exchange.

Pharmaceutical companies offer information resources through their websites and indication-related platforms, which often include recommendations for reputable knowledge sources, such as patient organizations. However, in relation to the central concerns of CML patients, it is recommended that comprehensive knowledge resources be developed specifically addressing individual medications, their side effects, and potential consequences of treatment failures. Frequently encountered problems and their solutions within therapy should also be addressed, bearing in mind that the solutions presented may be standard and may not apply to every individual case.

Interestingly, the desk research did not reveal any evidence of psychological support being provided by the pharmaceutical industry to CML patients, despite emotional and psychological needs being expressed in the forums. There is a clear need for improvement in this area by the pharmaceutical industry.

As patients increasingly discuss specific studies and research articles, involving them at an early stage can help incorporate patient-relevant parameters into studies from the outset, leading to an improved quality of life for patients. However, the skepticism and critical views expressed by patients towards the pharmaceutical industry are concerning. To better support patients, the pharmaceutical industry must allocate more resources to patient education and enhance their understanding of patient engagement activities. It is crucial to communicate that the pharmaceutical industry provides neutral and high-quality information in compliance with strict regulations. One suggestion is to utilize video platforms such as YouTube to deliver education and reach a wider audience.

In conclusion, the overall needs of CML patients are adequately addressed by the pharmaceutical industry. However, by considering patient needs more comprehensively, pharmaceutical companies have the opportunity to tailor their offerings to better meet patient expectations. There is room for improvement in various areas, particularly in response to the increasing demand for psycho-oncological support. Additionally, better and more comprehensive education, extending beyond general knowledge, is required regarding medications and their side effects. It is important to recognize that each patient has individual needs and expectations from the pharmaceutical industry, and the findings of this study may not fully capture these variations.

#### 4.2.2. Topic evolution

In addition, the development of the main topics over time was examined. The forum leukaemie-online.de shows that the topic “leukemia simple” has the greatest relevance on average from 2010 to 2022. The topic indicates an exchange within the community about the disease itself. Thus, the exchange about CML with other sufferers within a forum does not decrease over time. The topic “answer-hope” belongs to the least relevant topics and shows a value of zero in the months 01/2020 and 01/2022. The topic could mean that people ask questions in the forum and hope for feedback from members. The decrease in relevance could mean that the community is so active that forum members asking questions can always count on responses from the community, so there is no need to hope for a response. The topic “reply appreciate” of the forum of the website cmlsuport.org.uk also has the lowest average value and can be justified with the same statement. Also noticeable is the topic “dose mg”, which has been actively used since 2018, but has especially increased since 2020. This could be justified by the updated ELN recommendations from 2020, which reflect the new therapy procedure using TKIs. The previous version is from 2013. Furthermore, this could again point to the need to reduce the dosage of the drugs, if possible.

## 5. Limitations

While our study has yielded valuable insights, it is important to acknowledge its limitations. Firstly, it should be recognized that each patient has unique needs and expectations regarding the pharmaceutical industry and other stakeholders. Therefore, our findings may not encompass the full spectrum of these individual preferences.

Additionally, one limitation of our study is the inclusion of both German and English language sources for the analysis of user-generated content (UGC). This mixing of languages introduces the possibility that identified needs may be influenced by differences in healthcare systems across various countries. Moreover, we were unable to differentiate between patients from different countries of origin. Even though UGC in German may not necessarily represent the needs of patients exclusively residing in Germany, it could also pertain to other German-speaking countries. The same applies to English-speaking CML patients. Future research could address this limitation by considering country-specific factors when examining patient needs.

In light of these limitations, it is essential for further research to delve into the nuances of individual patient contexts and incorporate country-specific considerations. This would provide a more comprehensive understanding of the diverse needs and expectations of CML patients, enhancing the applicability and relevance of future interventions and support programs.

## 6. Conclusions

Over the past years, there has been a noticeable shift in the healthcare system, with patients assuming a more prominent role, particularly within the pharmaceutical industry. Patients are now equipped with a greater understanding of their illnesses and available treatment options, resulting in an increased emphasis on patient engagement and empowerment within the pharmaceutical sector. This paper aims to confirm the core concept that the pharmaceutical industry is undergoing a transformation, gradually adopting a position as a healthcare provider.

The pharmaceutical industry has begun to recognize the significance of prioritizing patient needs. The resources allocated to patient engagement in the context of CML are currently deemed adequate. However, continuous improvement and development remain crucial, primarily through direct patient engagement. Key areas of focus should include education about CML, medications, and fostering an environment for exchange and support within the CML community.

Furthermore, the pharmaceutical industry's goal of involving patients earlier in clinical trials aligns with the interests of patients. To investigate this hypothesis further, a targeted analysis should be conducted. This would provide valuable insights into the feasibility and potential benefits of patient inclusion in clinical trials.

Establishing trust is paramount for the pharmaceutical industry to effectively support patients in their journey of disease progression. Educating patients about the pharmaceutical industry's commitment to patient engagement is essential in building this trust. Trust and understanding serve as the foundation for successful and targeted support from the pharmaceutical industry.

In conclusion, the evolving landscape of the healthcare system has propelled patients to the forefront, prompting the pharmaceutical industry to adapt and adopt a role as a healthcare provider. Through continuous improvement, direct patient engagement, and fostering trust, the industry can effectively support patients in their battle against disease.

## Data Availability

Publicly available datasets were analyzed in this study. This data can be found here: https://github.com/data-for-health/CML-Topic-Models.
