# Supplementary figures and images for: Listen to the patients! Identifying CML patients' needs analyzing patient-generated content with AI-driven methodologies
Source: Front Digit Health. 2023 Dec 5;5:1243215. doi: 10.3389/fdgth.2023.1243215 (PMC10729659; doi:10.3389/fdgth.2023.1243215)

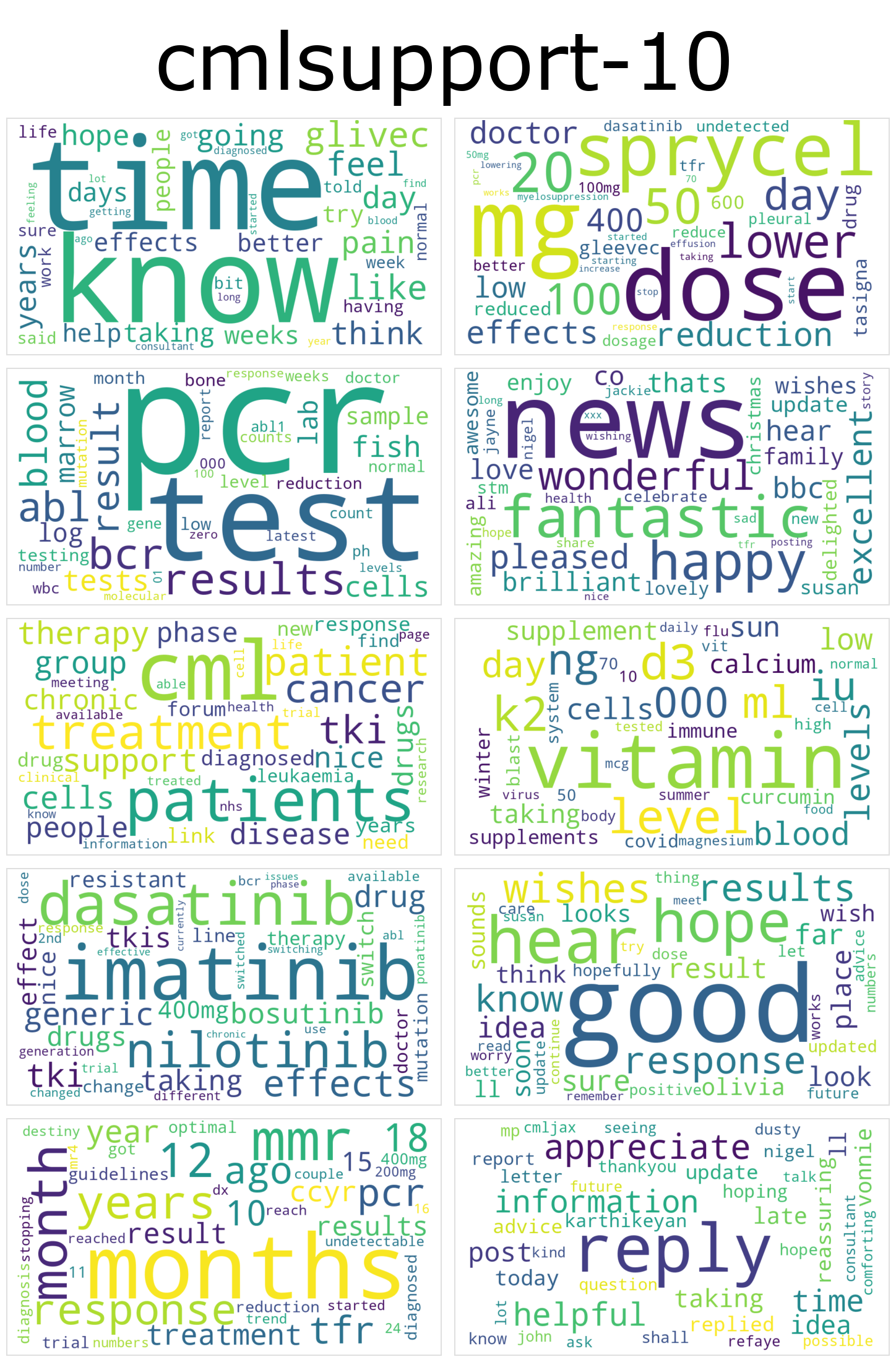

Supplement: Supplementary file 1 [file Datasheet1.zip › Data Sheet 1_v1/Image 1.PNG]

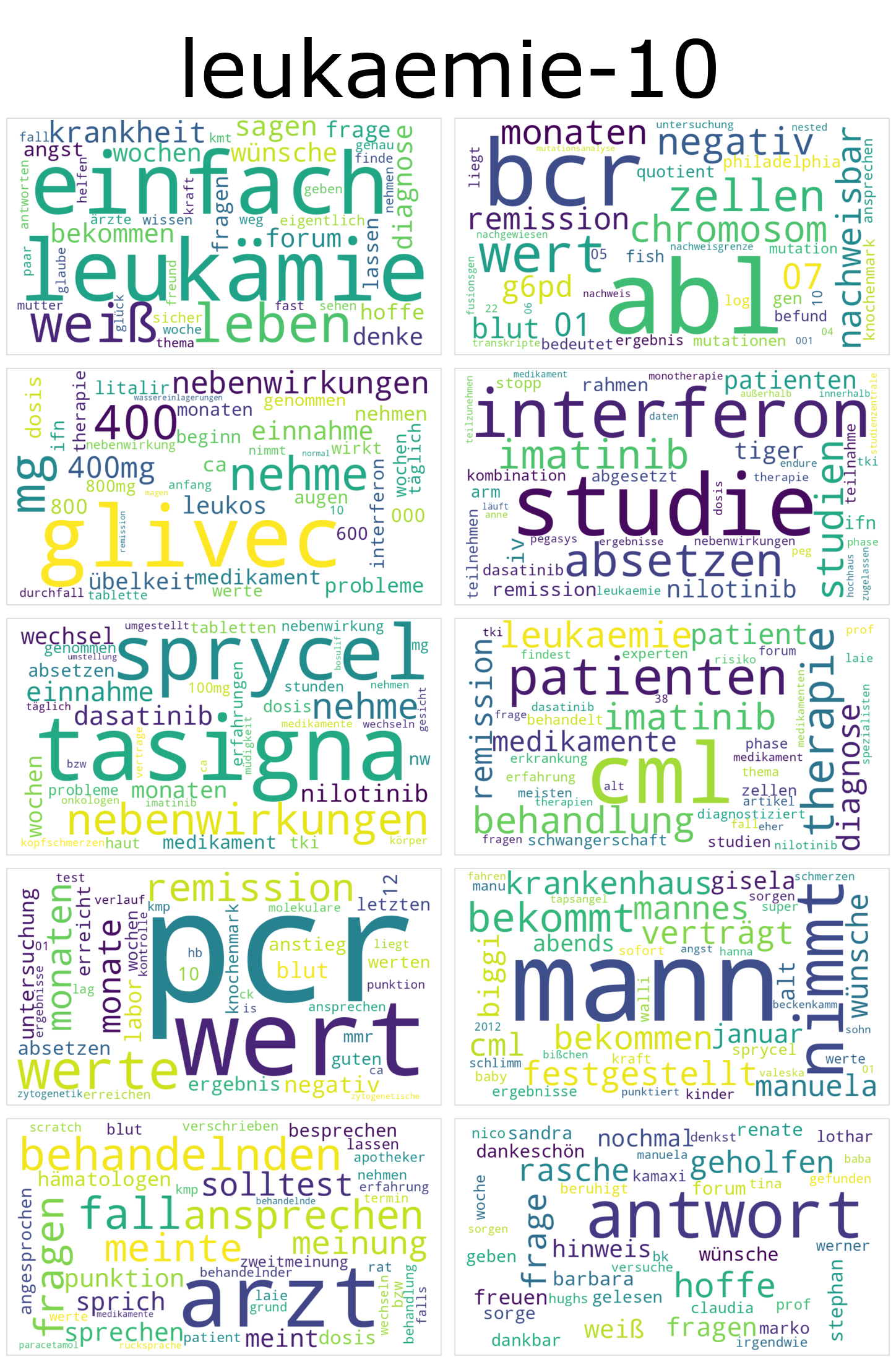

Supplement: Supplementary file 1 [file Datasheet1.zip › Data Sheet 1_v1/Image 3.PNG]

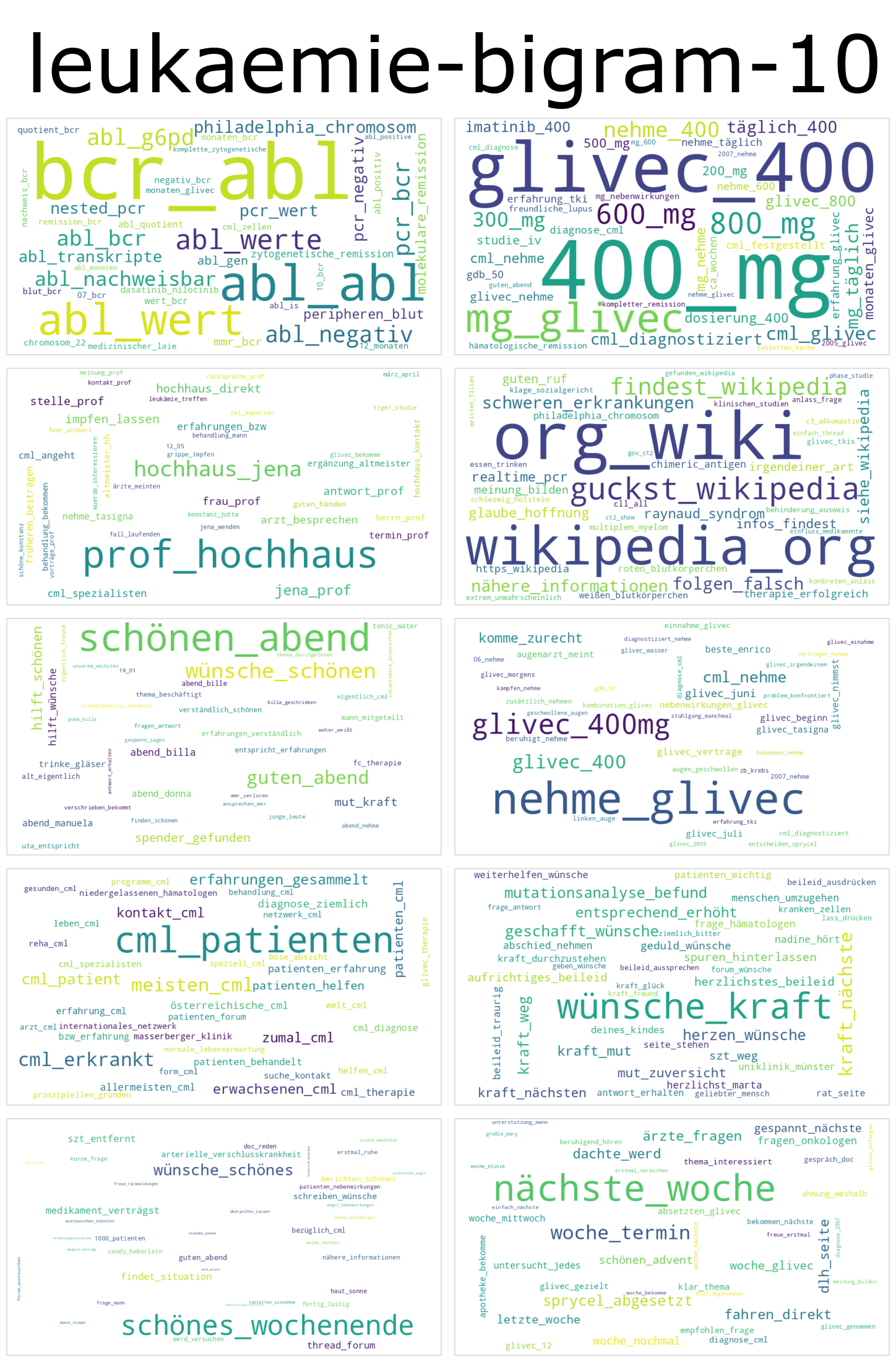

Supplement: Supplementary file 1 [file Datasheet1.zip › Data Sheet 1_v1/Image 4.PNG]

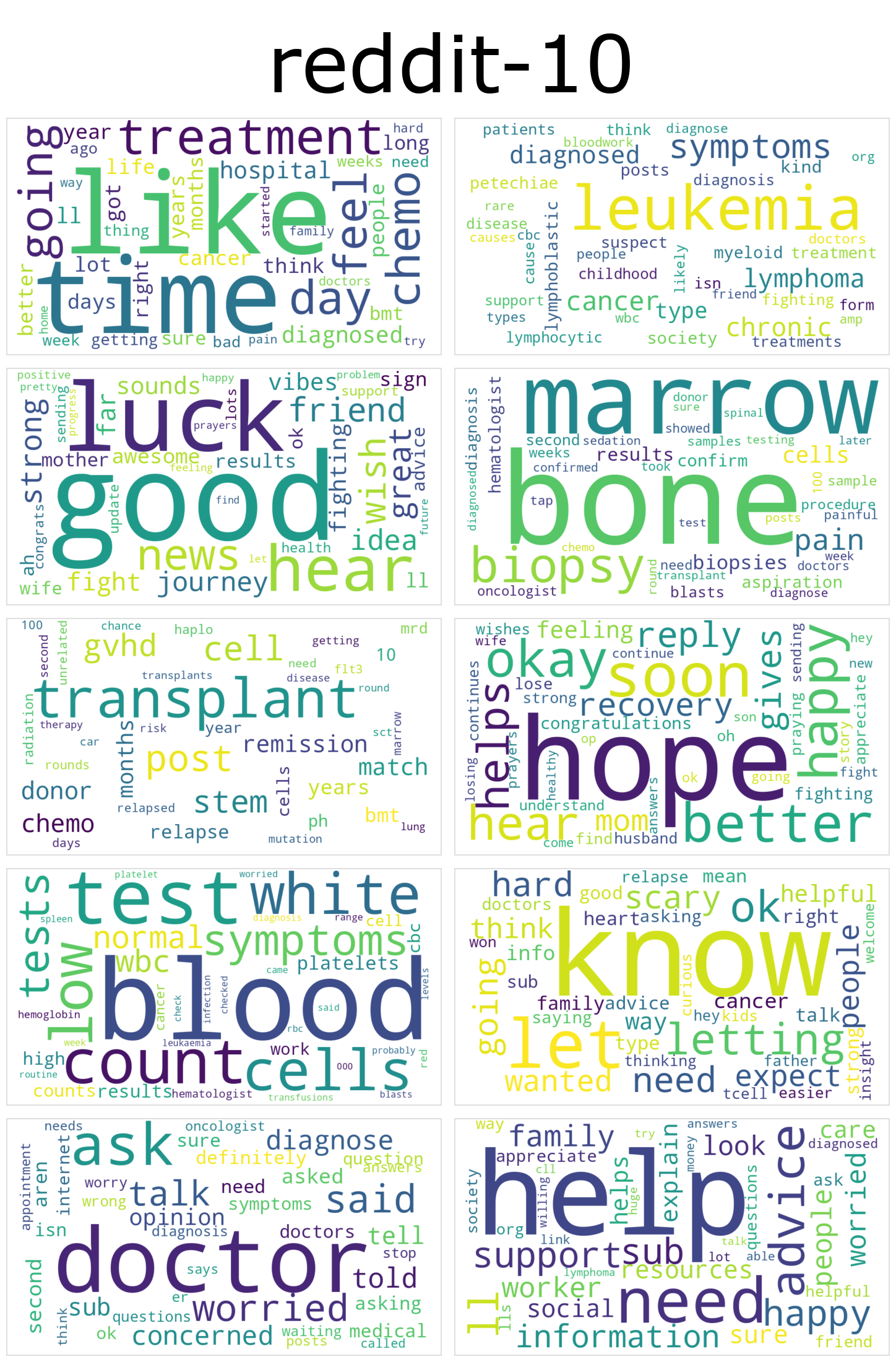

Supplement: Supplementary file 1 [file Datasheet1.zip › Data Sheet 1_v1/Image 5.PNG]

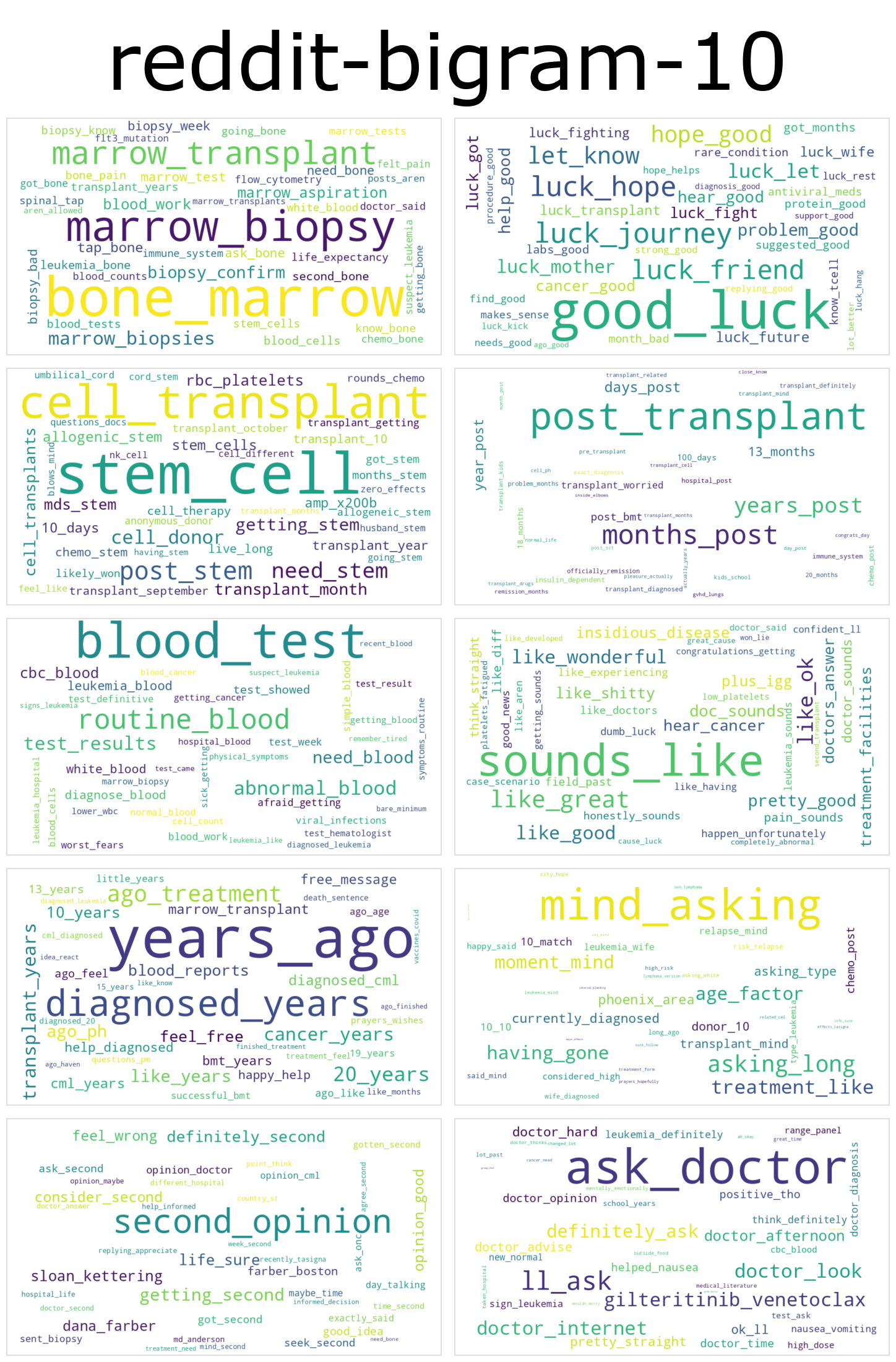

Supplement: Supplementary file 1 [file Datasheet1.zip › Data Sheet 1_v1/Image 6.PNG]
